# Phenotypic and Genotypic Study of Antibiotic Resistance *blaPER‐1*, *blaVEB*, and *blaSHV‐1* Genes in Clinical Isolates of *Pseudomonas aeruginosa*

**DOI:** 10.1002/hsr2.72329

**Published:** 2026-04-24

**Authors:** Milad Gholampour Matin, Reza Shapouri, Mohammadreza Nahaei, Mojtaba Mohammadi Roknabadi, Rasoul Shokri

**Affiliations:** ^1^ Department of Microbiology, Faculty of Basic Sciences, Zanjan Branch Islamic Azad University Zanjan Iran; ^2^ Department of Microbiology and Laboratory Sciences, Faculty of Medicine, Tabriz Medical Sciences Islamic Azad University Tabriz Iran

**Keywords:** antibiotic resistance, blaPER‐1 gene, blaSHV‐1 gene, blaVEB gene, extended spectrum beta‐lactamase, *Pseudomonas aeruginosa*

## Abstract

**Background and Aims:**

*Pseudomonas aeruginosa* is a non‐fermentative, opportunistic Gram‐negative bacterium that causes 10%–15% of nosocomial and burn wound infections worldwide. This bacterium is resistant to various antibiotics through multiple mechanisms, including acquisition of resistance genes. The aim of this study was to perform a phenotypic and genotypic analysis of *blaPER‐1*, *blaVEB*, and *blaSHV‐1* genes in clinical isolates of *P. aeruginosa*.

**Methods:**

One hundred and ten *P. aeruginosa* isolates were collected from clinical specimens. The pattern of antimicrobial resistance was determined using the disk diffusion method. Phenotypic analysis of ESBL production was performed using the combined disk method. Additionally, molecular identification of *blaPER‐1*, *blaVEB*, and *blaSHV‐1* β‐lactamase genes was carried out by polymerase chain reaction (PCR).

**Results:**

The highest resistance and sensitivity were observed against ceftazidime (86.36%) and colistin (78.18%), respectively. Among the 110 isolates, 72 were phenotypically ESBL‐producing. The *blaPER‐1* gene was detected in 33 isolates (45.83%), *blaVEB* in 18 isolates (25.00%), and *blaSHV‐1* in 27 isolates (37.50%).

**Conclusion:**

Overall, phenotypic analysis indicated that 65.45% of isolates were ESBL producers, and the presence of *blaPER‐1*, *blaVEB*, and *blaSHV‐1* genes was associated with increased resistance to ceftazidime, amikacin, ciprofloxacin, cefepime, and gentamicin. However, further studies are needed on ESBL‐producing isolates, as well as the identification of effective antibiotics for the treatment of *P. aeruginosa* infections.

## 1 Introduction

The Pseudomonadaceae family includes a large group of Gram‐negative bacilli, which are obligate aerobic, non‐fermenting, oxidase‐positive, motile, and able to grow in minimal environments. *Pseudomonas aeruginosa* is the main human pathogen in this group, although other Pseudomonas species rarely cause disease [1, 2]. This bacterium is an important pathogen and a cause of death in patients with cystic fibrosis, neoplastic diseases, and severe burns. *P. aeruginosa* can cause serious infections, including septicemia, pneumonia, endocarditis, otitis, and keratitis [3, 4].

Due to increasing antibiotic resistance, controlling the prevalence of *P. aeruginosa* is often difficult and can be a major factor in mortality among immunocompromised patients [5]. *P. aeruginosa* inherently resists many antimicrobial and antiseptic substances [6]. This resistance is partly due to the impermeability of its outer membrane, which limits the entry of antibiotics via passive or active transport [7]. Additionally, *P. aeruginosa* can acquire antibiotic resistance through several mechanisms, such as the production of beta‐lactamase and carbapenemase enzymes, overexpression of efflux pumps, and reduced membrane permeability [6, 7].

Beta‐lactam antibiotics are a major class of antibiotics containing a beta‐lactam ring, including penicillin, cephalosporins, monobactams, and carbapenems [8]. The production of beta‐lactamase enzymes by certain bacteria leads to hydrolysis of the beta‐lactam ring and loss of antibiotic activity [9, 10]. The increased use of new‐generation broad‐spectrum cephalosporins has driven the appearance of extended‐spectrum beta‐lactamases (ESBLs) [11]. Previous studies have identified numerous genes encoding metallo‐beta‐lactamase enzymes in Gram‐negative bacteria, especially *P. aeruginosa* [12, 13]. Consequently, the prevalence of multidrug‐resistant microorganisms has steadily increased and remains a major concern [14, 15].

Given the importance of resistance mechanisms in opportunistic microorganisms, particularly *P. aeruginosa*, and the destructive effect of metallo‐beta‐lactamase enzymes on carbapenems (imipenem and meropenem), this study aimed to investigate the phenotypic and genotypic presence of *blaPER‐1*, *blaVEB*, and *blaSHV‐1* genes in clinical isolates of metallo‐beta‐lactamase‐producing *P. aeruginosa*.

## 2 Materials and Methods

### 2.1 Selection of Isolates

In this prospective study, 110 clinical isolates of *P. aeruginosa* were collected from blood, urine, tracheal tube, and burn wound samples of patients referred to the microbiology lab at Asadabadi Hospital, Tabriz, Iran, between January 2020 and December 2021. The study was approved by the Ethics Committee of the Zanjan Branch, Islamic Azad University (Zanjan, Iran). All patients were informed about the study and provided written informed consent prior to participation. The sample size was calculated using the formula (*n* = *Z* ^2 ^× *P *× (1 − *P*)/*d* ^2^) with a 95% confidence level, an expected prevalence of ESBL‐producing *P. aeruginosa*, and a 5% margin of error. The isolates detection was performed using standard biochemical and microbiological tests, including Gram staining, oxidase, catalase, hemolysis, indole, Voges‐Proskauer, citrate utilization, urease, and hydrogen sulfide (H₂S) production tests. To preserve the confirmed isolates for subsequent analyses, the bacteria were stored in Tryptic Soy Broth (TSB) supplemented with 15% glycerol.

### 2.2 Antibiotic Susceptibility Testing

Antimicrobial susceptibility of the identified *P. aeruginosa* isolates was investigated by the disk diffusion method according to the Clinical and Laboratory Standards Institute (CLSI; 2021) on Muller–Hinton agar. Several antibiotic discs, such as gentamicin (10 µg), imipenem (30 µg), ceftazidime (30 µg), cefepime (30 µg), piperacillin (100 µg), ciprofloxacin (5 µg), amikacin (30 µg), tobramycin (5 µg), and colistin (10 µg), were used for antimicrobial susceptibility tests. *P. aeruginosa* ATCC 27853 was used as a control.

### 2.3 Phenotypic Detection of ESBL β‐Lactamases

Isolates showing simultaneous resistance to three or more classes of antibiotics were classified as multidrug‐resistant (MDR). The isolates were tested for production of ESBL by the combination disc method using ceftazidime (30 µg), ceftazidime‐clavulanic acid (30 µg–10 µg), cefotaxime (30 µg), and cefotaxime–clavulanic acid (30–10 μg) antibiotic discs. An isolate was considered ESBL‐positive if the diameter of the inhibition zone around the combined disc was ≥ 5 mm larger than that of the disc alone [16].

### 2.4 Genotypic Detection of ESBL β‐Lactamases

Genomic DNA was extracted from the isolates using a commercial kit (Invitek Stratec Business, Canada) following the manufacturer's instructions. The quality of the extracted DNA was investigated by electrophoresis on 1% agarose gel. Moreover, the quantity of the extracted DNA was investigated according to the OD 260/280 ratio. The extracted genomic DNA samples were stored at −20°C for polymerase chain reaction (PCR) reactions. The used primers are presented in Table 1. Finally, the obtained PCR products were electrophoresed on 2% agarose gel. *P. aeruginosa* ATCC 27853 was used as a control [16].

**Table 1:** The sequences and characteristics of the primers used in the study.

Gene	Sequence	Product size	Reference
*blaPER‐1*	5′‐ATGAATGTCATTATAAAAGCT‐3′	927	[17]
	5′‐TTAATTTGGGCTTAGGG‐3′		
*blaVEB*	5′‐CGACTTCCATTTCCCGATGC‐3′	1000	[18]
	5′‐GGACTCTGCAACAAATACGC‐3′		
*blaSHV‐1*	5′‐TCAGCGAAAAACACCTTG‐3′	471	[19]
	5′‐TCCCGCAGATAAATCACCA‐3′		

### 2.5 Statistical Analysis

Statistical analysis was conducted using SPSS software (version 16) and Microsoft Excel (2010). Associations between the presence of *blaPER‐1*, *blaVEB*, and *blaSHV‐1* genes and antibiotic resistance were evaluated using *χ*² and Fisher's exact tests. A *p* value < 0.05 was considered statistically significant.

## 3 Results

### 3.1 Bacterial Isolates

Out of 622 specimens submitted to the microbiology laboratory, 110 isolates were identified and confirmed by biochemical tests. Among these, 23 isolates were obtained from blood samples, 27 from urine samples, 26 from tracheal aspirates, and 34 from burn wound samples.

### 3.2 Antibiotic Sensitivity

In this study, the resistance patterns of *P. aeruginosa* isolates against a panel of nine antibiotics from different classes were investigated. The results of antimicrobial susceptibility tests indicated that resistance was most prevalent against tobramycin (76.36%), ciprofloxacin (80.00%), and ceftazidime (86.36%). In contrast, the highest susceptibility rate was observed for colistin (78.18%) (Table 2).

**Table 2:** Antibiotic resistance of our *P. aeruginosa* isolates.

Antibiotics	Abbreviation	Dose (µg)	Resistance patterns		
			Sensitive	Semi‐sensitive	Resistant
Ceftazidime	CAZ	30	8 (7.27%)	7 (6.36%)	95 (86.36%)
Ciprofloxacin	CIP	5	20 (18.18%)	2 (1.81%)	88 (80.00%)
Tobramycin	TOB	10	19 (17.27%)	7 (6.36%)	84 (76.36%)
Cefepime	CEP	30	21 (19.01%)	6 (5.45%)	83 (75.45%)
Piperacillin	PIP	100	36 (32.72%)	2 (1.81%)	72 (65.45%)
Imipenem	IPM	30	51 (46.36%)	9 (8.18%)	50 (45.45%)
Gentamicin	GEN	10	71 (64.54%)	3 (2.72%)	36 (32.72%)
Amikacin	AMK	30	72 (65.45%)	5 (4.55%)	33 (30.00%)
Colistin	CST	10	86 (78.18%)	4 (3.63%)	20 (18.18%)

### 3.3 Phenotypic Detected ESBL‐Producing Isolates

The phenotypic detection of ESBL‐producing isolates showed that among 110 isolates, 72 (65.45%) isolates were ESBL producers. Of these 72 ESBL‐producing isolates, 17 (out of 23) were obtained from blood specimens, 17 (out of 27) from urine specimens, 14 (out of 26) from tracheal aspirate specimens, and 24 (out of 34) from burn wound specimens (Table 3).

**Table 3:** The frequency of ESBL‐producing isolates collected from different clinical specimens.

Clinical specimens	ESBL positive	ESBL negative	Total
Blood	17 (15.45%)	6 (5.45%)	23 (20.90%)
Urine	17 (15.45%)	10 (9.09%)	27 (24.55%)
Tracheal tube	14 (12.72%)	12 (10.90%)	26 (23.63%)
Burn wound	24 (2.18%)	10 (9.09%)	34 (30.90%)
Total	72 (65.45%)	38 (34.55%)	110 (100%)

### 3.4 Genotypic Detected ESBL‐Producing Isolates

The genotypic detection of ESBL‐producing isolates showed that the frequency of *blaPER‐1*, *blaVEB*, and *blaSHV‐1* genes were 45.83% (33 isolates), 75.00% (54 isolates), and 37.50% (27 isolates), respectively (Table 4). In addition, 42 isolates (58.33%) were positive for more than one ESBL gene, demonstrating co‐occurrence of multiple resistance genes in these strains.

**Table 4:** The frequency of *blaPER‐1*, *blaVEB*, and *blaSHV‐1* genes in ESBL‐producing *P. aeruginosa* isolates.

Gene	Clinical specimens	Positive	Negative	Total
*blaPER‐1*	Blood	2 (2.78%)	15 (20.82%)	17 (23.61%)
	Urine	6 (8.33%)	11 (15.28%)	17 (23.61%)
	Tracheal aspirate	8 (11.11%)	6 (8.33%)	14 (19.44%)
	Burn wound	17 (23.61%)	7 (9.72%)	24 (33.33%)
	Total	33 (45.83%)	39 (54.17%)	72 (100%)
*blaVEB*	Blood	3 (4.16%)	14 (19.44%)	17 (23.61%)
	Urine	4 (5.55%)	13 (18.05%)	17 (23.61%)
	Tracheal aspirate	3 (4.16%)	11 (15.28%)	14 (19.44%)
	Burn wound	8 (11.11%)	16 (22.22%)	24 (33.33%)
	Total	18 (25.00%)	54 (75.00%)	72 (100%)
*blaSHV‐1*	Blood	3 (4.16%)	14 (19.44%)	17 (23.61%)
	Urine	9 (12.50%)	8 (11.11%)	17 (23.61%)
	Tracheal aspirate	8 (11.11%)	6 (8.33%)	14 (19.44%)
	Burn wound	7 (9.72%)	17 (23.61%)	24 (33.33%)
	Total	27 (37.50%)	45 (62.50%)	72 (100%)

### 3.5 Association of ESBL Genes and Antibiotic Resistance

The presence of the blaPER‐1 gene was significantly correlated with resistance to ceftazidime and amikacin. In addition, statistical analysis revealed a significant relationship between blaVEB gene presence and resistance to ciprofloxacin, cefepime, and gentamicin. Furthermore, the presence of the blaSHV‐1 gene was significantly associated with resistance to ceftazidime and ciprofloxacin (Table 5).

**Table 5:** The association between the presence of *blaPER‐1*, *blaVEB*, and *blaSHV‐1* genes and antibiotic resistance in *P. aeruginosa* isolates.

Antibiotic	Resistant isolates (*n* = 72)	Presence of *blaPER‐1*	*p* value	Presence of *blaVEB*	*p* value	Presence of *blaSHV‐1*	*p* value
Ceftazidime	68 (94.44%)	51	0.023	29	0.364	55	0.005
Ciprofloxacin	63 (87.50%)	31	0.188	49	0.044	54	0.019
Tobramycin	57 (79.16%)	31	0.110	26	0.139	31	0.088
Cefepime	55 (76.38%)	28	0.092	41	0.003	32	0.199
Piperacillin	54 (75.00%)	27	0.089	31	0.055	21	0.222
Imipenem	54 (75.00%)	31	0.055	33	0.051	29	0.146
Gentamicin	39 (54.16%)	30	0.011	13	0.277	19	0.287
Amikacin	38 (52.77%)	23	0.112	31	0.010	12	0.340
Colistin	22 (30.55%)	12	0.236	9	0.211	7	0.571

## 4 Discussion

The intrinsic resistance of *P. aeruginosa* to various antimicrobial agents causes difficulty in its prevention and treatment. *P. aeruginosa* employs several mechanisms to develop resistance [5], among which the production of ESBLs is one of the most common [8]. Metallo‐beta‐lactamases are a class of beta‐lactamase enzymes that lead to resistance against most beta‐lactam antibiotics. The global increase in hospital‐acquired infections is partly due to the rapid proliferation of ESBL‐producing bacteria, among which *P. aeruginosa* is a key contributor [20]. After *Staphylococcus aureus* and *Escherichia coli*, *P. aeruginosa* is identified as the third leading cause of nosocomial infections [21].

In a similar study in Isfahan, Fazeli et al. reported that 94% of *P. aeruginosa* isolates were resistant to imipenem, piperacillin, and ciprofloxacin, while all isolates (100%) showed resistance to ceftazidime and ticarcillin [22]. Ranjbar et al., in a study conducted in Tehran, found that 100% of *P. aeruginosa* isolates obtained from burn wounds exhibited multidrug resistance. They also found high resistance rates to imipenem (97.5%), amikacin (90%), piperacillin (87.5%), ceftizoxime (72.7%), gentamicin (68.5%), ciprofloxacin (65%), cefepime (60%), and ceftazidime (57.0%) [23]. Previous studies have suggested a significant association between the extensive use of antibiotics such as imipenem, ceftazidime, ciprofloxacin, and amikacin, and the increased resistance of *P. aeruginosa* [24, 25]. High resistance rates of *P. aeruginosa* to nalidixic acid (86.54%) and ceftazidime (79.81%) were reported by Salehi et al. [25]. Similarly, Mihani et al. found that 71% of *P. aeruginosa* isolates were resistant to ceftazidime [26]. Fallah et al., in a separate study, found that 83% of *P. aeruginosa* isolates exhibited resistance to imipenem, with 48% producing ESBLs [27]. The resistance rates observed in these studies were higher than those reported in European and North American countries, but were comparable to rates reported in South America, Africa, and neighboring countries such as India and Pakistan [28, 29]. These differences may be attributed to variations in geographic location, sample size, and sampling methods.

The prevalence of ESBL‐producing *P. aeruginosa* isolates was reported as 34%, 39%, and 40% in various studies conducted in Iran by Shakibaie et al. [30], Shahcheraghi et al. [31], and Mirsalehian et al. [32], respectively. The higher frequency of ESBL‐producing isolates observed in the present study may be attributed to the increased use of broad‐spectrum cephalosporins in our region.

Phenotypic detection of ESBL‐producing *P. aeruginosa* isolates may sometimes yield false results due to various resistance mechanisms, such as low membrane permeability and the presence of efflux pumps. Therefore, molecular investigation of beta‐lactamase genes can be helpful. Currently, several types of these enzymes have been identified in *P. aeruginosa* strains, including CTX, SHV, PER, GES, TEM, OXA, and VEB [3]. Matar et al. reported a 21% prevalence of the SHV gene in their study conducted in Lebanon [33]. Similarly, Jiang et al. in China reported the prevalence of *blaVEB‐1*, *blaTEM‐1*, and *blaOXA‐10* genes as 23.28%, 17.80%, and 16.43%, respectively [34]. The frequent presence of beta‐lactamase genes in clinical isolates may result from horizontal gene transfer among bacteria.

The observed association between ESBL genes and resistance to antibiotics outside the beta‐lactam group may be explained by co‐resistance, as ESBL genes are often carried on plasmids or other mobile genetic elements that harbor additional resistance determinants. For example, plasmids carrying *blaSHV* or *blaTEM* genes can also encode resistance to aminoglycosides or fluoroquinolones, which may account for the simultaneous resistance observed. Furthermore, selective pressure from the use of various antibiotics can favor the survival and propagation of multidrug‐resistant isolates, enhancing the likelihood of encountering resistance to multiple antibiotic classes. These findings underscore the importance of monitoring ESBL‐producing *P. aeruginosa* not only for beta‐lactam resistance but also for multidrug resistance, which has significant implications for treatment strategies and infection control.

One limitation of our study is that we focused only on *blaPER‐1*, *blaVEB*, and *blaSHV‐1* genes, while other ESBL genes such as *blaTEM*, *blaCTX‐M*, and *blaOXA* are also prevalent and may contribute to antibiotic resistance in *P. aeruginosa*. These three genes were selected based on their reported predominance in our study region, but we acknowledge that resistance mediated by other genes may have been overlooked. Additionally, we did not perform sequencing of the detected genes to identify possible point mutations or variants that could have arisen from the parent enzymes, which limits the understanding of genetic diversity among isolates. Finally, phylogenetic analysis was not conducted in this study, so the evolutionary relationships between the ESBL genes could not be assessed. Future studies, including a broader panel of ESBL genes, sequencing, and phylogenetic analysis, are recommended to provide a more comprehensive understanding of resistance mechanisms in *P. aeruginosa*.

## 5 Conclusions

Overall, phenotypic investigation of antibiotic resistance demonstrated that 65.45% of clinical *P. aeruginosa* isolates from clinical specimens were ESBL producers. Our findings suggest that the presence of *blaPER‐1*, *blaVEB*, and *blaSHV‐1* genes may be associated with resistance to ceftazidime, amikacin, ciprofloxacin, cefepime, and gentamicin. Given the high frequency of ESBL‐producing strains, preventing unnecessary antibiotic use and identifying multidrug‐resistant *P. aeruginosa* isolates may be useful for the management of hospital infections.

## Author Contributions

**Milad Gholampour Matin:** conceptualization, methodology, investigation, data curation, formal analysis, validation, writing – original draft. **Reza Shapouri** and **Mohammadreza Nahaei:** conceptualization, supervision, methodology, project administration, writing – review and editing. **Mojtaba Mohammadi Roknabadi:** methodology, investigation, data curation, validation, writing – original draft. **Rasoul Shokri:** software, formal analysis, data curation, validation, methodology, writing – review and editing, funding acquisition.

## Funding

The authors have nothing to report.

## Ethics Statement

This study was approved by the Ethics Committee of Zanjan Branch, Islamic Azad University (Zanjan, Iran).

## Consent

All patients were informed and signed an informed consent form before participating in this study.

## Conflicts of Interest

The authors declare no conflicts of interest.

## Transparency Statement

The corresponding author, Reza Shapouri, affirms that this manuscript is an honest, accurate, and transparent account of the study being reported; that no important aspects of the study have been omitted; and that any discrepancies from the study as planned (and, if relevant, registered) have been explained.

## Data Availability

Data will be made available on request.
